# CoQ10 Supplementation in Patients Undergoing IVF-ET: The Relationship with Follicular Fluid Content and Oocyte Maturity

**DOI:** 10.3390/antiox7100141

**Published:** 2018-10-13

**Authors:** Stefano Raffaele Giannubilo, Patrick Orlando, Sonia Silvestri, Ilenia Cirilli, Fabio Marcheggiani, Andrea Ciavattini, Luca Tiano

**Affiliations:** 1 Department of Clinical Sciences, Polytechnic University of Marche, 60123 Ancona, Italy; i.cirilli@pm.univpm.it (I.C.); ciavattini.a@libero.it (A.C.); 2 Department of Life and Environmental Sciences, Polytechnic University of Marche, 60131 Ancona, Italy; p.orlando@univpm.it (P.O.); s.silvestri@univpm.it (S.S.); f.marcheggiani@univpm.it (F.M.); l.tiano@univpm.it (L.T.)

**Keywords:** Coenzyme Q10, In vitro fertilization-embryo transfer (IVF-ET), follicular fluid, oocyte maturity

## Abstract

Background: The target of the reduced fecundity with aging is the oocyte. The follicular fluid and its components are strongly linked with the environment of the maturing oocyte. The aim of the present study was to evaluate CoQ10 bioavailability in follicular fluids after oral supplementation and its possible implication in oocyte maturation. Methods: Fifteen female partners of infertile couples, aged 31–46, undergoing IVF-ET and taking 200 mg/day oral CoQ10 were compared to unsupplemented patients. CoQ10 content, its oxidative status and total antioxidant capacity were evaluated also in relation to oocyte maturation indexes. Results: CoQ10 supplementation produced a significant increase in follicular content and a significant improvement of its oxidative status. Follicular fluid total antioxidant capacity highlighted a significant decrease in patients supplemented with CoQ10, specially in women >35 years. CoQ10 supplementation was associated with a significant decrease in total antioxidant capacity of fluid from follicles containing mature oocyte, moreover CoQ10 oxidative status was also significantly reduced but in follicles containing immature oocyte. Conclusions: Our observation leads to the hypothesis that the oral supplementation of CoQ10 may improve follicular fluid oxidative metabolism and oocyte quality, specially in over 35-year-old women.

## 1. Introduction

During the past decades, cultural and social changes produced a significant delay in reproductive age in many cases [1] related to a decrease in response to ovarian stimulation, reduced embryo quality and pregnancy rate and an increased incidence of miscarriages and fetal aneuploidy. The lack of an age-related decline in pregnancy outcome, when donor oocytes are used, suggests that the target of the reduced fecundity with aging is the oocyte. Moreover, there is a well-established association between late maternal age and an increased risk for oocyte and embryo anomalies [2,3]. The follicular fluid and its components are strongly linked with the environment of the maturing oocyte and ageing-associated oxidative stress has been shown to interfere with the complex follicular microenvironment impairing maturation [4]. Oxidative damage might damage endogenous follicular components and alter follicular fluid composition dueto abnormal sieving of plasma lipoprotein at the blood-follicular barrier [5]. Follicular fluids have been reported to contain only High-density-lipoproteins (HDL), however recent studies highlighted the presence of Apo B proteins associated to low, very low and intermediate density protein (LDL, VLDL and IDL) [4]. Levels of these lipoproteins and their oxidized forms usually increase in follicular fluids with ageing and in obese patients [6]. Moreover, in metabolically active granulosa cells mitochondrial dysfunction might contribute to oxidative stress, cell damage and apoptosis [7]. Mitochondria are a sensitive site for oxidative damage being both a source and a target of reactive oxygen species (ROS), conditions associated with infertility, such as ageing that might exacerbate this process [8].

In order to counterbalance oxidative insults, follicles present several lines of defenses constituted by low molecular weight antioxidants, associated with lipoproteins and enzymatic antioxidants produced by granulosa cells. In this respect, animal studies suggest that antioxidant supplementation is beneficial to overcome deleterious effects of stress induced ROS on mouse oocytes [9]. This process is enhanced by a transudation of mediators from the circulation into the follicular fluid. In women undergoing invitro fertilization (IVF), there is a notable imbalance in the ovarian follicular fluid environment that is closely linked to oxidative stress. This has been shown to adversely affect oocyte and embryo development and eventually pregnancy outcome [10].

Coenzyme Q10, mainly known for its bioenergetics role as a proton and electron carrier in the inner mitochondrial membrane, is also an endogenous lipophilic antioxidant, ubiquitous in biological membranes [11] and transported in plasma mainly by LDL and HDL where together with vitamin E prevents lipoprotein oxidation. Coenzyme Q10 was also shown to affect gene expression by modulating the intracellular redox status [12]. In reproductive biology, CoQ10 has been shown to play a role in fertility of both males and females [13]. In seminal plasma and sperm cells of infertile men with idiopathic asthenozoospermia, we reported lowered levels and increased oxidation of CoQ10 [14]. The administration of CoQ10 in these patients was shown to increase CoQ content and improve its oxidative status as well as semen kinetic features [15,16]. In the female reproductive system early reports on CoQ10 content, in various rat tissues after exogenous supplementation, showed a remarkable uptake from the adrenal gland and the ovary. The total CoQ10 content of these organs more than doubled, while other organs did show only modest increases [17]. A study from Stoikovic et al. [18] has looked into the effect of CoQ10 as a supplement for the in vitro culture of bovine embryos and found a significantly higher rate of early embryo cleavage, blastocyst formation rate, hatching rate, percentage of expanding blastocysts and a larger size of the inner cell mass (ICM). In the same paper the authors observed also an increased ATP content in the group of embryos cultured with CoQ10. All of those parameters suggest improved embryo quality. In line with this observation, more recently Ben-Meir et al. have shown that CoQ10 restores oocyte mitochondrial function and fertility during reproductive ageing [19], highlighting the key role of coenzyme Q10 in reproductive ageing by using animal models with deficient CoQ10 synthesis and showing decreased ovarian reserve that could be rescued by CoQ10 supplementation. In human clinical studies we evaluated CoQ10 content in follicular fluid in relation to oocyte fertilization and embryo grading [20]. CoQ10 levels resulted significantly higher in mature versus dysmorphic oocytes. Similarly, levels resulted in significantly enhanced grading I–II versus grading III–IV embryos. This data was recently confirmed by Akarsu S. et al. [21] and could highlight a role of CoQ10 in protecting follicular lipoproteins from oxidation, preserving their functionality. Moreover, different studies have pointed out the ability of Coenzyme Q10 supplementation, 600 mg/day for 60 days, in improving the ovarian response in women with decreased ovarian reserve [22,23], consistently pointing out at treatment, leading to an improved responsiveness to follicular pre-stimulation in terms of lower gonadotropin requirement during IVF. Notably to our knowledge, no human studies verified the bioavailability of CoQ10 to the follicular milieu and related to its biological effects. In the present study, we aimed at verifying whether CoQ10 oral supplementation, in concentration considered in the European Union as dietary supplement namely 200 mg/day for 30 days, resulted in an elevation of CoQ10 follicular fluid content. Moreover, we also evaluated whether increases in CoQ10 follicular content were associated with increase in number of mature oocyte. Finally, improvement of oocyte quality was evaluated measuring nuclear maturation status, the morphology of the cytoplasm and on the appearance of the extra-cytoplasmic structures.

## 2. Materials and Methods

### 2.1. Patients Selection and Study Design

The study was conducted on 30 patients undergoing IVF–ET program in the Obstetrics and Gynecology Department, “Salesi University Hospital” of Polytechnic University of Marche, Ancona (Italy) (Table 1).

All patients underwent a medical screening, including history and clinical examination, presented a clinical history of primary infertility between 24 and 48 months. Before the data collection, the protocol of this study was approved Departmental Review Board, all women were informed before the procedure and they provided their written consent. The study was conducted in accordance with the Declaration of Helsinki with the internal protocol code A12. All patients recruited had both ovaries and regular menstrual cycles every 27 to 32 days and normal ovulatory function as formally proved by biphasic basal body temperature, mid luteal phase plasma progesterone levels and ultrasonographic scanning. All patients were of Caucasian race, aged 31–46 years, had normal blood pressure and body mass index (BMI), were non-smoking and not taking any medication within the 3 months before enrollment in the present study and were not involved in intensive exercise. Infertility diagnosis included tubal disease and idiopathic infertility while, patients with anatomic abnormalities of the genital tract such as uterine anomaly, fibroids, ovarian cyst, pelvic or systemic inflammatory disease were excluded from the study. All women recruited were requested to follow their usual diet to avoid effects due to variable CoQ10 intake in food. Fifteen patients agreed to take the same formulation of CoQ10 (Myoquinone, Pharmanord, Copenhagen, Denmark), 200 mg/day in two daily administrations, with main meals for 30 days. The remaining 15 patients, among those not usually taking CoQ10 agreed to participate to the study as a control group. CoQ10 treatment preceded pituitary desensitization, and proceeded until the oocyte pick-up for an average period lasting 30–35 days.

### 2.2. Follicular Stimulation

Controlled ovarian hyperstimulation was performed by a standard protocol and at time of oocytes aspiration, the follicular fluid was sampled. Oocytes were recovered by transvaginal ultrasound-guided follicle aspiration. A minimum of 2 and up to 5 oocytes and follicular fluids for each woman were collected for a total of 120 follicular fluids (69 FF samples from CoQ supplemented women and 51 FF samples in controls). In each patient, the follicular fluid (FF) was individually aspirated in conjunction with oocyte retrieval. Selection criteria was in chronological order during the procedure. The follicular fluid was aspirated without the contamination of flushing medium and new pipettes being used for each aspiration to eliminate contamination. At each collection time, the volume of fluid and the presence of oocytes were recorded for each follicular fluid sample. After oocyte isolation, the follicular fluid was centrifuged at 3000× for 10 min at 4 °C to remove debris, blood and granulosa cells and was then frozen at −80 °C until assayed. Follicular fluids that were contaminated with significant quantities of blood cells were not used for analysis. After the oocyte retrieval, oocyte classification was performed. The maturational status of the oocytes was recorded taking into account nuclear maturation status, the morphology of the cytoplasm and on the appearance of the extracytoplasmic structures. In particular, the presence of the first polar body (PBI) and a meiotic spindle (MS) were used as indicators of mature, Metaphase II (MII) stage oocytes [24].

### 2.3. Follicular Fluids CoQ10 Levels

CoQ10 levels were assayed in follicular fluids fractions as described by Silvestri S. et al. [25], with a single dilution step after extraction of 50 μL of FF with 250 μL of propanol and vigorously mixed with vortex. A peculiarity of the system was the use of a post-separation reducing column (Shiseido CQR; 20 × 2.0 mm) capable of fully reducing the peak of oxidized CoQ10. Follicular fluids levels of total CoQ10 was expressed as μg/mL. Since CoQ10 is mainly vehiculated in lipoproteins values were also normalized for total cholesterol and expressed as nmol CoQ10/mmol Chol.

### 2.4. ORAC Assay of Follicular Fluids

The Oxygen Radical Absorbance Capacity (ORAC) fluorescence assay was performed in FF using a microplate reader (Synergy HT, BIO TEK, Vinooski, VT, USA) as previously described [26] with slight modifications. Briefly, FF samples were diluted 1/50 in PBS 75 mM and added to a solution of fluorescein 0.08 µM. Fluorescence decay was initiated by AAPH (1.8 mM final concentration) radical attack following decomposition at 37 °C. Fluorescence kinetic was monitored for 210 min with readings every 2 min. A standard curve to compare results was constructed using Trolox in the reaction, a short chain analogue of vitamin E as a standard. The ORAC activity of the samples was calculated using Gen5 software and expressed as millimoles Trolox equivalents (mMTeq).

### 2.5. Statistical Analysis

Sample size was calculated taking into account a minimally significant expected increase of 35% in follicular CoQ10 content, expressed in nmol CoQ10/mmol cholesterol, whose measured within group standard deviation based on 20 patient observations that was about 36 [20]. 15 subjects were providing an 80% chance that an 0.05 level test of significance will find a statistically significant difference between two sample means. The various biological parameters relative to IVF cycles for these two groups of patients were compared by Mann–Whitney U test. Significance of the correlation was estimated using Pearson correlation coefficient and sampling distributions were verified using chi-square test. Median and quartile values of each tested parameter were calculated and presented as box plots where the line in the center of box, the box and the bars represent respectively the median, 50% and 25% of the measurements for each parameter. In the text data are reported as mean ± SEM.

## 3. Results

### 3.1. Follicular Coenzyme Q10 Content and Oxidative Status

**Figure 1:**
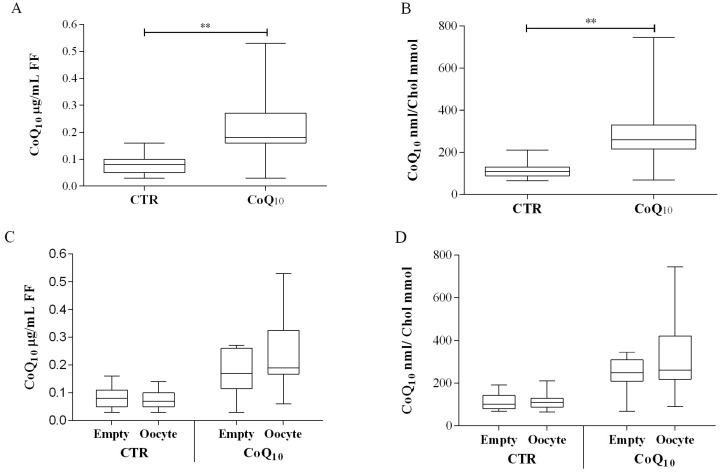
Follicular fluid (FF) coenzyme Q10 content in control (CTR*n* = 51) and supplemented IVF patients (CoQ10*n* = 69). CoQ10 is expressed both per volume of follicular fluid as µg CoQ10/mL FF (**A**) or normalized for cholesterol content as nmol CoQ10/mmol Chol (**B**). Sub analysis in relation to oocyte content as µg CoQ10/mL FF (**C**) or normalized for cholesterol content nmol CoQ10/mmol Chol (**D**). ** *p* < 0.01. (oocyte containing follicles *n* = 30 CTR; *n* = 44CoQ).

CoQ10 follicular fluid content in control patients was 0.08 ± 0.03 µg/mL. Supplementation of patients for 30–35 days with 200 mg/day CoQ10, produced a significant increase in follicular content to 0.22 ± 0.13 µg/mL (+280%, *p* < 0.01) (Figure 1A).

**Figure 2:**
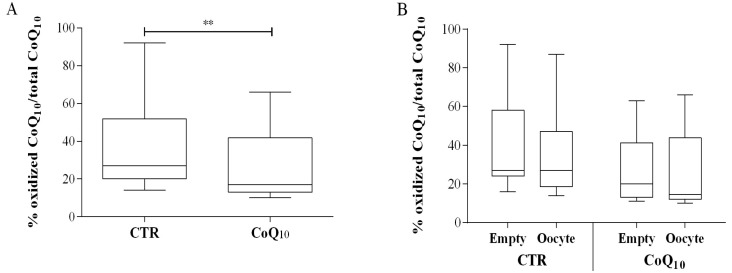

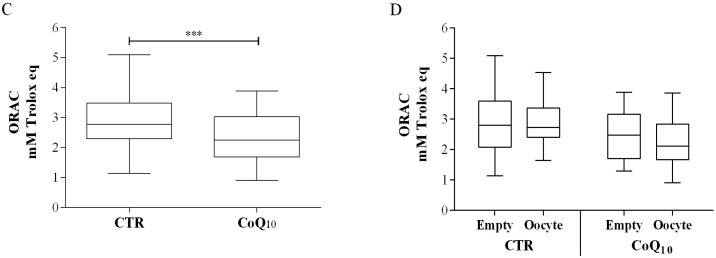
Follicular fluid (FF) coenzyme Q10 oxidative status and Oxygen Radical Absorbing Capacity (ORAC) in control (CTR*n* = 51) and supplemented IVF patients (CoQ10*n* = 69). CoQ10 oxidation is expressed as % of oxidized CoQ10/total CoQ10 (**A**,**B**), while ORAC value is expressed as mMTrolox equivalents (**C**,**D**). Sub analysis in relation to oocyte content as % of oxidized CoQ10/total CoQ10 (**B**) or mMTrolox equivalents (**D**). ** *p* < 0.01; *** *p* < 0.001. (oocyte containing follicles *n* = 30 CTR; *n* = 44CoQ).

This increase was significant also after cholesterol normalization producing a variation from 113 ± 37 to 312 ± 188 nmol CoQ10/mmol Cholesterol (+166%; *p* < 0.01) (Figure 1B). Considering the number of oocyte in each analyzed follicle, in subjects not supplemented with CoQ10 no significant differences were observed between follicles containing at least one oocyte (and follicles from which no oocytes were recovered (Figure 1C,D). On the contrary, in supplemented subjects, follicle containing oocytes presented higher levels of CoQ10 compared to follicles from which an oocytes was not recovered expressed both as µg/mL FF (values 0.25 ± 0.14 vs. 0.18 ± 0.08 µg/mL respectively) and after cholesterol normalization as CoQ10 nmol/Chol mmol, (values 351 ± 222 vs. 246 ± 78 nmol/mmol respectively) almost reaching significance levels (*p* = 0.058) (Figure 1C,D). In relation to oxidative parameters, the percentage of oxidized CoQ10 was significantly lower in patients supplemented with CoQ10 (27 ± 18%) compared to control (38 ± 24%), *p* < 0.01 (Figure 2A).

No significant variations in CoQ10 oxidative status were observed in both groups in relation to oocyte content (Figure 2B). Interestingly FF total antioxidant capacity measured by means of the ORAC assay, highlighted a significant decrease in patients supplemented with CoQ10 in relation to control group (from 2.9 ± 0.9 to 2.4 ± 0.7 mMTrolox eq.; *p* < 0.001) (Figure 2C). Also in this case no significant variations were observed in both groups in relation to oocyte content (Figure 2D). CoQ10 supplementation resulted in a slight and non-significant increase in the percentage of oocyte-containing follicles from 57% in the control group to 63% in the supplemented group (data not shown).

### 3.2. Sub Analysis of Oocyte-Containing Follicles

**Figure 3:**
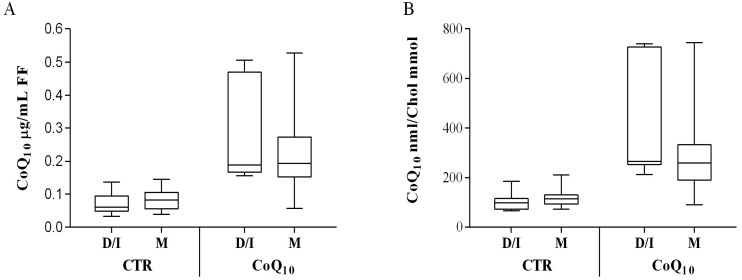

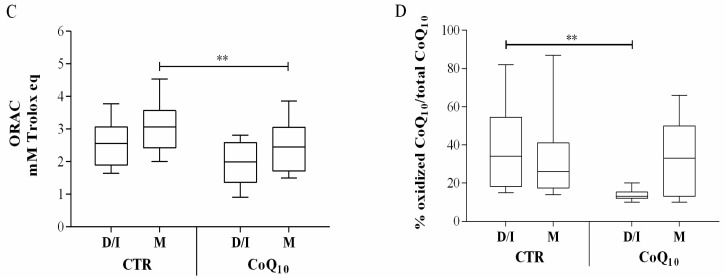
Oocyte-containing follicles coenzyme Q10 content and oxidative parameter in control (CTR) and supplemented IVF patients (Q10) in relation to follicle maturation stage. CoQ10 is expressed both per volume of follicular fluid as µg CoQ10/mL FF (**A**) or normalized for cholesterol content as nmol CoQ10/mmol Chol (**B**); ORAC value is expressed as mMTrolox equivalents (**C**) while CoQ10 oxidation is expressed as % of oxidized CoQ10/total CoQ10 (**D**). ** *p* < 0.01.

Comparison of follicular fluid from follicle containing oocyte, did not show any significant difference in CoQ10 content in relation to maturation stage of oocyte, in samples from untreated patients. On the contrary, in CoQ10 treated subjects, a different distribution in follicular content was observed in association with mature oocyte (M) that were homogeneously distributed close to the median values (25° percentile 0.17 µg/mL of FF, 75° percentile 0.27 µg/mL of FF) (Figure 3A). On the contrary dysmorphic and immature oocyte (D/I) showed a broader distribution characterized by several samples with high CoQ10 follicular content (25° percentile 0.17 µg/mL, 75° percentile 0.44 µg/mL (Figure 3A). This data was more pronounced after cholesterol normalization, nonetheless it did not reach statistical significance. (Figure 3B). Also, in relation to the antioxidant capacity, follicle containing mature oocyte (M) were different from dysmorphic and immature oocyte (D/I). Independently from treatment, ORAC values were higher in mature oocyte (M) (Figure 3C). CoQ10 supplementation was associated with a significant decrease in total antioxidant capacity of FF from follicles containing mature oocyte (M) from 2.52 ± 0.79 to 2.26 ± 0.71 mMTrolox eq.; *p* < 0.01 (Figure 3C). Moreover, CoQ10 oxidative status was also significantly affected in follicles containing immature oocyte (D/I) (*p* < 0.01), while no significant changes were observed in follicles containing mature oocyte (M) (Figure 3D).

### 3.3. Fertilization Rate and Embryo Grading

As reported in Table 2, 88% of mature oocyte were successfully fertilized in the CoQ10 group (22 out of 25) and 74% (20 out of 27) in the control group (CTR). Embryos were graded all in class I (82%) and II (18%) in the CoQ10 group while, in the control group also grade III embryos were observed (15%) while I grade embryo were 60%.

### 3.4. Age Related Considerations

**Figure 4:**
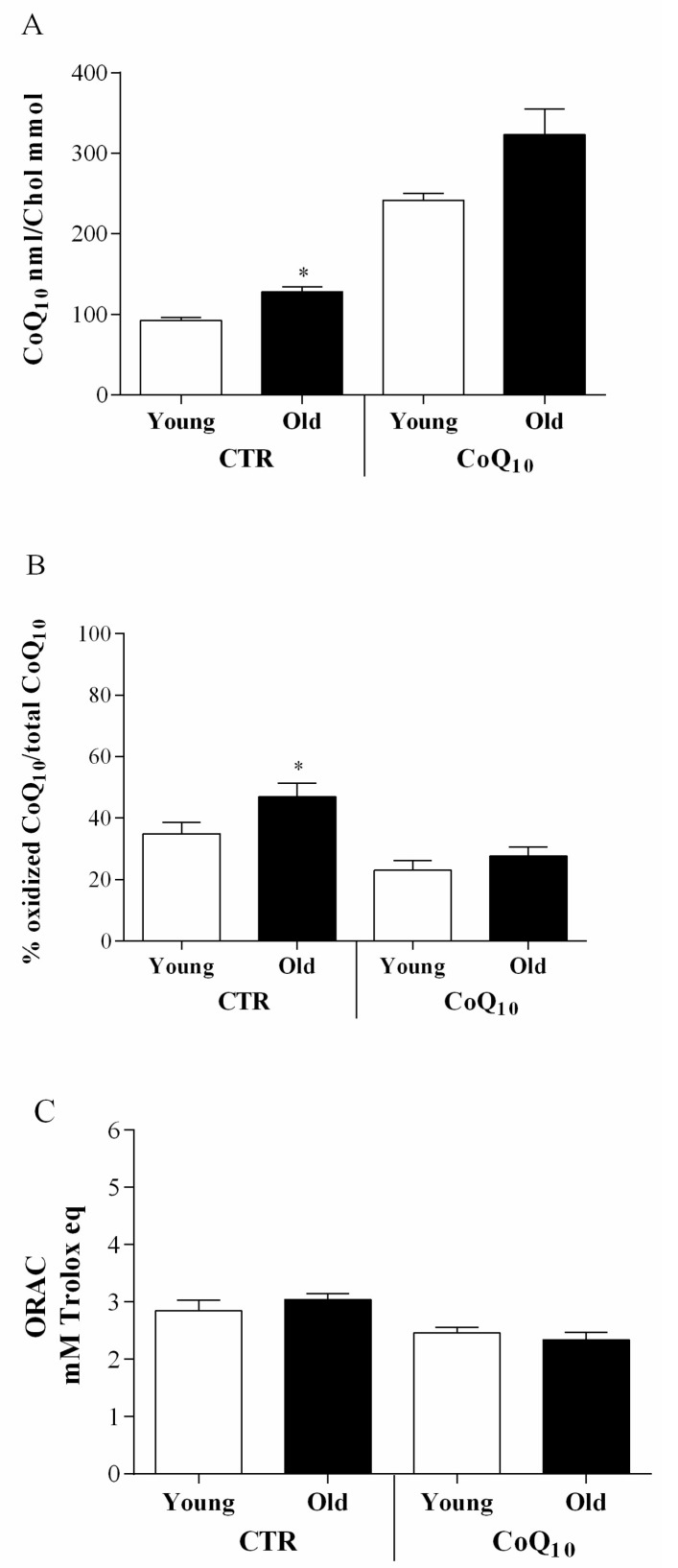
Age related comparison of CoQ10 concentration expressed as nmol CoQ10/mmol Chol (**A**) and oxidative parameters such as % of oxidized CoQ10/total CoQ10 (**B**) and ORAC mMTrolox equivalents (**C**) values, in untreated (CTR) and supplemented (CoQ10) IVF patients below 35 years (Young) or above 35 years (Old). * *p* < 0.05.

In follicles from which an oocyte was not recovered or hosting immature oocyte, from untreated patients, a positive correlation with age was observed for CoQ10 nmol/cholesterol mmol (r = 0.32; *p* < 0.05), total antioxidant capacity (r = 0.47; *p* < 0.01) and percentage of oxidized CoQ10 (r = 0.54; *p* < 0.01). In CoQ10 treated patients, the same type of follicles showed a single positive correlation between age and percentage of oxidized CoQ10 (r = 0.48; *p* < 0.01).In particular, comparing base line CoQ10 follicular content, in younger (<35 years of age) and older (>35 years of age) patients, a significant increase was observed in the latter (*p* < 0.05) (Figure 4A), in particular in its oxidized form (*p* < 0.05) (Figure 4B). Moreover, in this age-group a slight though not significant increase in total antioxidant capacity was also observed (Figure 4C). None of these age-related differences were observed in the CoQ10 treated group. In particular, CoQ10 levels were remarkably higher in both age groups and this increase was associated with a significantly lower content in oxidized CoQ10 as well as a lower FF total antioxidant capacity.

## 4. Discussion

Developing oocytes within the ovarian follicle grow rapidly and require an active supply of energy and cholesterol [26]. Nutrients and signaling molecules are contained in a peculiar microenvironment constituted by the surrounding follicular fluid. FF play a fundamental role for the oocyte and embryo developmental potential [27]. Although ROS have a functional role during embryo development, excess of reactive oxygen species may alter the composition of this microenvironment impairing fertility [28,29]. Therefore, follicles are properly and dynamically equipped to withstand oxidative stress, providing a tight coordination of enzymatic and low molecular weight antioxidants levels [30]. Deregulation of these processes, often observed in obese and older women, result in harmful oxidative stress associated with ovarian disorders and an-ovulation [6,31]. Among the effectors and regulators of oxidative stress in the follicular fluids attention should be drawn on lipoprotein composition and mitochondrial activity in the oocyte and granulosa cells. In particular, the production of energy for the metabolic requirement of the oocyte is provided solely by mitochondria that are present in remarkable number in mature oocytes, much higher than other cell types with elevated cellular requirements such as muscle cells and neurons [8,32]. The oocyte mitochondrial pool remains unaltered up to 40 years before follicle maturation, therefore from a “mitochondrial senescence” point of view these cells present similarities with non-replicative cells such as neural cells. During follicular recruitment mitochondrial biogenesis peaks reaching a striking ratio 1:2 of mtDNA to total DNA content. Oocyte mitochondrial mutation and impaired energy production increase with age and these failures are transmitted to the whole mitochondrial pool of the mature oocyte. This phenomenon might underlie increased oxidative stress and abnormal embryonic development. In this frame nutrition is known to play a role in the modulation of reproductive performances [8,33], nonetheless our understanding of the role of micronutrients, trace elements and vitamins are still lacking. In this study we evaluated the effect of oral administration of Coenzyme Q10, an endogenous lipophilic mitochondrial nutrient with important antioxidant activity in the lipoprotein environment. We have previously shown, for the first time, that CoQ10 is present in follicular fluids and its endogenous levels and oxidative status correlate with embryo grading in IVF-ET patients [20]. Here we demonstrate, for the first time, that oral intake of CoQ10 at dietary supplement dosage (200 mg/day) results in a significant increase in FF of CoQ10 in its active reduced form. When compared patients of different age groups (below and above 35 years of age), these enhancements provided a significant protection in relation to age-related biochemical modifications in follicular fluid parameters, that could be associated both to its antioxidant activity in lipoproteins and its bioenergetic role. The follicular fluid contains a mixture of components transferred from blood plasma or produced by the metabolism of granulosa cells, providing over 200 proteins essential for follicular development [4]. Major functions of these proteins can be summarized in promotion of maturation of the oocyte, protection of the follicle from toxic injury and oxidative stress and lipid transport. Coenzyme Q10 is vehiculated in plasma by lipoproteins that reasonably represent the carrier of exogenous CoQ10 in the FF. Lipoproteins are deeply involved in all the above mentioned functions and play an essential role in oocyte maturation and reproductive success. This is also confirmed by the fact that, dyslipidemia is associated with impaired or abrogated fertility in different pathologies and animal models lacking lipoprotein receptor. Hence, it is reasonable that the enhanced protection of lipoproteins by coenzyme Q10 might support oocyte maturation and reproductive success.The peculiar composition in proteins of FF is determined by a selection at the blood-follicle barrier, constituted by thecal blood capillaries that act as a hormone-sensitive molecular sieve. During the periovulatory period the basal membrane becomes permeable to serum proteins up to 300 KDa and as a result of this sieving effect, in normal conditions, LDL and VLDL content is rather scarce in FF and the main lipoprotein component is a selected HDL population, primarily HDL3 [34]. This accounts for differences in lipid composition depleted in cholesterol esters and enriched in surface phospholipids such as sphingosine-1-phosphate, an active mediator of angiogenesis and mitogenesis. Both these processes are essential in the development of the ovarian follicle and its subsequent transition to corpus luteum [35]. Moreover, HDL associated proteins, including PON-1, exert an important cytoprotective effect on the oocyte and surrounding granulosa cells [36]. The central role of this lipoprotein led to the definition of an hepatic-ovarian HDL associated axis of fertility [34]. Although coenzyme Q10 is mainly transported in LDL, HDL are able to remarkably increase their basal content upon oral administration and CoQ10 content has been shown to strongly correlate with PON-1 activity in isolated HDL [37]. Blood-follicle is a dynamic barrier responsive to hormonal status and therefore composition of FF changes during maturation also due to granulosa cell secretions. This explains the presence in low amounts of LDL and oxidized LDL, recently observed in FF [4,31,38]. Alteration of the blood-follicular barrier permeability might also help explaining by the apparently counter-intuitive increase of CoQ10 in follicular fluid, in un-supplemented older subjects. In fact, an enhanced permeability to LDL, the main carriers of CoQ10, might result in a significant increase in CoQ10 content. Moreover, dysregulation of filtering systems at the basal level, induction of compensatory antioxidant defenses in granulosa cells or passive release of cellular content by apoptotic cells, might result in an increase in antioxidant content that is not functionally active but rather the manifestation of adverse effects in follicular maturation. In fact, several studies report inverse associations between antioxidant content and reproductive success [6,27,33,39,40]. In this respect, the ageing process has been shown to affect antioxidant content and total antioxidant capacity in many cases. In particular, the concentration of enzymatic antioxidants has been shown to increase with age [41]. The efficacy of this increase is rather limited and it has been proposed to be even deleterious since an increase in superoxide dismutase (SOD), not associated with equal increases in peroxidases, leads to an accumulation of H_2_O_2_ [42]. These evidences could explain the observed increase in ORAC level in older subjects as well as its decrease following CoQ10 supplementation. In this frame, the antagonistic effect of CoQ10 might be interpreted as an effect in preserving follicular homeostasis.

## 5. Conclusions

Our results highlight different beneficial effects associated with coenzyme Q10 follicular fluid elevation following oral administration, that might support the supplementation of subjects in late reproductive status. Observed biochemical endpoints should be complemented in an ad hoc study with relevant clinical endpoints. In particular, quantitative assessment of the percentage of mature oocyte as well as number of pregnancies deserves attention and should be verified in a larger placebo controlled randomized study.

## Figures and Tables

**Table 1:** Characteristics of patients enrolled.

PARAMETERS	CoQ_10_ Group	CTR Group
AGE (years)	31–41 (32.7 mean)	32–46 (34.6 mean)
BMI	21.1	20.9
INFERTILITY (months)	24–48	24–48
N° CYCLES	15	15
FSH	1600–2100	1750–2300

**Table 2:** Fertilization rate and embryo grading in the CoQ10 group compared to untreated control (CTR). EG-I (Embryo grading I: cells are of equal size; no fragmentation seen), EG-I (Embryo grading II: cells are of equal size; minor fragmentation only), EG-III (cells are of unequal size; no fragmentation to moderate fragmentation).

GROUPS	*Fertilization rate*	*EG-I*	*EG-I*	*EG-III*
*CoQ_10_*	22/25 (88%)	18 (82%)	4 (18%)	-
*CTR*	20/27 (74%)	12 (60%)	5 (25%)	3 (15%)
